# Intact FGF23 concentration in healthy infants, children, and adolescents, and diagnostic usefulness in patients with X-linked hypophosphatemic rickets

**DOI:** 10.1007/s40618-023-02202-4

**Published:** 2023-11-22

**Authors:** G. I. Baroncelli, M. R. Sessa, C. Pelosini, S. Bertelloni, A. Michelucci, B. Toschi, P. Piaggi, D. Peroni, P. Comberiati

**Affiliations:** 1 Pediatric and Adolescent Endocrinology, Division of Pediatrics, Department of Obstetrics, Gynecology and Pediatrics, University Hospital, Pisa, Italy; 2 Chemistry and Endocrinology Laboratory, Department of Laboratory Medicine, University Hospital, Pisa, Italy; 3 Unit of Molecular Genetics, Department of Laboratory Medicine, University Hospital, Pisa, Italy; 4 Section of Medical Genetics, Department of Medical and Oncological Area, University Hospital, Pisa, Italy; 5 National Institute of Diabetes and Digestive and Kidney Diseases, National Institutes of Health, Phoenix, AZ USA; 6 Department of Information Engineering, University of Pisa, Pisa, Italy; 7 Department of Clinical and Experimental Medicine, Section of Pediatrics, University of Pisa, Pisa, Italy

**Keywords:** Adolescents, Children, Fibroblast growth factor 23, Infants, X-linked hypophosphatemic rickets

## Abstract

**Objective:**

FGF23 measurement may have a diagnostic role to investigate patients with phosphate disorders. However, normal values for infants, children, and adolescents have not been defined.

**Methods:**

In a total of 282 (males 145, females 137) healthy infants (*n* = 30), prepubertal (*n* = 147), pubertal (*n* = 59), and postpubertal (*n* = 46), and in twenty patients with X-linked hypophosphatemic rickets (XLH, age 10.2 ± 5.6 years) serum phosphate (automated analyzer), and plasma intact FGF23 (immunochemiluminescent sandwich assay, DiaSorin) concentrations were measured.

**Results:**

Intact FGF23 concentrations were higher in healthy infants than in prepubertal (*P* < 0.01) and postpubertal subjects (*P* < 0.05); pubertal subjects showed higher values (*P* < 0.05) than postpubertal subjects. Serum phosphate concentrations were higher (*P* < 0.001) in healthy infants than in prepubertal, pubertal, and postpubertal subjects. Pubertal subjects had higher (*P* < 0.001) serum phosphate concentrations than postpubertal subjects. Intact FGF23 and serum phosphate concentrations did not differ (*P* = NS) by sex, age of menarche, and time after menarche. In healthy subjects, there was no correlation between intact FGF23 and serum phosphate concentrations. Intact FGF23 concentrations were higher (*P* < 0.0001) in patients with XLH than in healthy subjects according to chronological age and pubertal development. In all patients, intact FGF23 concentrations were above 40 pg/mL; intact FGF23 concentrations were inversely correlated with serum phosphate concentrations (*r* = −0.65; *P* < 0.01).

**Conclusion:**

In healthy subjects, chronological age and puberty were main determinants of intact FGF23 concentrations. Intact FGF23 concentrations may be a useful marker for the early diagnosis of XLH in pediatric patients.

## Introduction

Fibroblast growth factor 23 (FGF23) is a 251 amino-acid peptide that is mostly secreted by osteocytes and osteoblasts. Its secretion is stimulated by high phosphate intake, hyperphosphatemia, 1,25-dihydroxyvitamin D (1,25(OH)_2_D), and parathyroid hormone (PTH). FGF23 has a crucial role in the regulation of phosphate metabolism by downregulating the sodium phosphate cotransporters in the proximal tubule, thus reducing phosphate reabsorption. FGF23 also decreases the expression of CYP27B1 and increases the expression of CYP24A reducing the production of 1,25(OH)_2_D and, consequently, the intestinal phosphate absorption. Moreover, FGF23 inhibits PTH synthesis and secretion [1–5].

Some FGF23 hyperfunction disorders leading to hypophosphatemia have been identified in which rickets/osteomalacia is a main clinical manifestation. They may be acquired, as in tumor-induced osteomalacia due to tumors secreting FGF23, or due to genetic mutations associated with loss-of-function of *PHEX*, *DMP1* and *ENPP1*, and gain-of-function of *FGF23* [6–8]. The absence of the active form of FGF23 due to an inactivating mutation in the *FGF23* or lack of FGF23 effect due to a mutation in the Klotho gene can cause hyperphosphatemia, hyperostosis and/or tumoral calcinosis [9].

X-linked hypophosphatemic rickets (XLH, MIM 307800) is the most common inherited form of rickets, with a prevalence of 1:20,000–60,000 [10, 11]. XLH is due to loss-of-function mutations in the *PHEX* gene, expressed in osteocytes and odontoblasts, which results in excess circulating FGF23. The typical presentation of patients with XLH includes the hallmarks of rickets and osteomalacia, progressive bowing deformities of the lower limbs, stunted growth with disproportionate short stature, bone pain, and physical dysfunction with reduced ability to perform daily activities [7, 12, 13]. Moreover, the majority of patients with XLH suffer dentinal and periodontal abnormalities causing recurrent periapical abscesses with fistulae [14–16].

FGF23 measurement may be a useful diagnostic tool to investigate patients with phosphate disorders [4, 6, 7]. However, normal reference values for intact or C-terminal FGF23 greatly diverged in children according to the method used and the range of age of the examined subjects [17–21].

In this study, intact FGF23 plasma concentrations in healthy infants, children, and adolescents were measured by the DiaSorin Liaison® XL assay to assess normal reference values according to chronological age and pubertal development; age of menarche and time after menarche were also assessed. Moreover, we evaluated the usefulness of intact FGF23 plasma concentration measurement for the early diagnosis in patients with XLH.

## Subjects and methods

### Healthy subjects

A total of 282 (145 males and 137 females) healthy Caucasian subjects, aged 1 month–19 years, observed as outpatients for routine investigation, preoperative assessment for minor surgeries, or screening blood tests were randomly recruited from our Department of Pediatrics at the University Hospital. Subjects with recent infections or co-morbidity were excluded. All the subjects were evaluated by the research group and were subdivided into 4 groups according to chronological age, pubertal stage, and sex: infants, age 1–11 months (*n* = 30; males *n* = 15, females *n* = 15); prepubertal (Tanner stage 1, *n* = 147; males *n* = 85, age 1–9 years; females *n* = 62, age 1–8 years); pubertal (Tanner stage 2–5, *n* = 59; males *n* = 21, age 10–16 years; females *n* = 38, age 9–14 years; postpubertal (Tanner stage 5, *n* = 46; males *n* = 24, age 17–19 years; females *n* = 22, 15–17 years). Nine pubertal females had not reached menarche yet and corresponded to a Tanner stage 2. Twenty-nine pubertal females (Tanner stages 3–5) had menarche at the mean age of 12.4 ± 0.7 years. All the postpubertal females had reached menarche. Time after menarche in postpubertal females was 4.3 ± 0.5 years.

An extensive and accurate clinical history was obtained by interview with the subject's parents or by the subject alone, as appropriate. All subjects were of normal length and weight at birth and did not take drugs known to affect bone or mineral metabolism. There was no history of any chronic disease or bone and mineral disorders.

### Patients with XLH

Twenty Caucasian patients with XLH (9 males and 11 females) aged 10.2 ± 5.6 years were recruited from our Endocrine Unit of Pediatrics at the University Hospital. The diagnosis of XLH was established by clinical, biochemical, and radiographic criteria, and was confirmed by *PHEX* gene mutation. Age at diagnosis was 2.3 ± 1.9 years (range 0.08–5.9 years). Sporadic *PHEX* gene mutation was demonstrated in eight patients (40%) and familial inheritance in twelve (60%). Karyotype was 46, XY in all males and 46, XX in all females. All patients were of normal weight and length at birth and had normal renal and liver function. All but one patient underwent conventional treatment with inorganic oral phosphate salts (doses titrated up to 32–45 mg elemental phosphorus/kg/d, in four–six divided doses) as Joulie’s solution (*n *= 3) or tablets (Reducto Spezial®, Temmler Pharma/Hormosan Pharma, Germany; *n* = 16) associated with alphacalcidol or calcitriol (doses titrated up to 21.5–35 ng/kg/d, once a day for alphacalcidol, *n* = 5; and in two or three divided doses for calcitriol, *n* = 14) from the time of diagnosis. All treated patients discontinued the conventional treatment 20 days before being enrolled in the study to reduce the effect of therapy on FGF23 production [22]. One patient was newly diagnosed and was not undergoing treatment at the time of FGF23 assessment.

### Study design

In all healthy infants, children, and adolescents, as well as in patients with XLH, fasting serum phosphate and intact FGF23 plasma concentrations were measured. In addition, in patients with XLH serum total alkaline phosphatase (ALP), PTH, and 1,25(OH)_2_D concentrations, and maximum rate of renal tubular reabsorption of phosphate normalized to the glomerular filtration rate (TmP/GFR) were assessed.

In the healthy subjects, the relationship between serum phosphate concentration, intact FGF23 plasma concentration, and chronological age or sex was calculated. In addition, in healthy postpubertal females the relationship between intact FGF23 plasma concentration and time after menarche was evaluated.

In patients with XLH the relationship between the biochemical parameters and chronological age was assessed. Moreover, the values of intact FGF23 in patients with XLH were compared with those of healthy subjects according to age and pubertal stage.

The study protocol was approved by the ethics committee for human investigation of the Department of Obstetrics, Gynecology and Pediatrics of the University Hospital. All parents of both patients and healthy subjects provided informed consent to participate in the study. The study was conducted according to the Declaration of Helsinki II and the Good Clinical Practice guidelines.

### Methods

In both healthy subjects and patients with XLH chronological age, recumbent length (≤ 2 years) or height (> 2 years) was measured by an infantometer device and by a calibrated wall-mounted Harpenden stadiometer, respectively, and expressed as Z-score according to Freeman et al. [23]. Pubertal stage was established according to the method of Tanner et al. [24]. In both healthy subjects and patients with XLH, postpuberty was arbitrarily defined as the association of a Tanner stage 5 with a chronological age from 17 to 19 years in males, and from 15 to 17 years in females. All the postpubertal healthy subjects and patients with XLH showed a near final height with an annual increment less than 1 cm/year [25].

Serum phosphate, creatinine, and ALP concentrations, as well as urinary phosphate and creatinine values were assessed within 1 h from sampling by an automated analyzer. Plasma EDTA samples for the measurement of intact FGF23 and intact PTH, and serum samples for 1,25(OH)_2_D, were separated within 2 h from sampling and stored at -20 °C until assayed; all the measurements were performed within 7 days from sampling.

Intact FGF23 plasma concentration was assessed by immunochemiluminescent sandwich assay developed by DiaSorin (Saluggia, Italy) on the Liaison® XL platform. Briefly, the method is based on three monoclonal antibodies: one coated on micro-particles and directed against the N-terminal portion of the intact FGF23 molecule; a second antibody labeled with fluoresceine, directed against the C-terminal portion, and a third antibody, bound with isoluminol, directed against fluoresceine. Accurate description and validation of this automated immunoassay have been reported; the method has been approved for clinical use by the European Community authorities [26, 27]. To validate the reference range measurement of intact FGF23 plasma concentration in healthy subjects, the suggested guidelines of the CLSI C28-A3 document were followed [28].

Intact PTH plasma concentration was measured by immunochemiluminescent two-step sandwich method that uses two monoclonal antibodies (Liaison® 1–84 PTH Assay, DiaSorin, Saluggia, Italy).

Serum 1,25(OH)_2_D concentration was measured by radioimmunoassay (^125^I, Immunodiagnostic Systems Holdings Ltd, UK).

In children and adolescents with XLH, urine samples for the calculation of TmP/GFR ratio were obtained from the second voluntary voiding in the morning and collected no more than two hours after previous voiding. In the infants with XLH, a single urine sample was collected in a clean container at any time (random/spot). The TmP/GFR was calculated as filtered phosphate per 100 ml GF excreted phosphate per 100 ml glomerular filtration by the following equation: Pp—(Up x PCr/UCr), where GFR was measured as creatinine clearance and Pp, Up, PCr, and UCr refer to the serum and urinary concentrations of phosphate and creatinine, respectively, as suggested by Stark et al. [29] and Brodehl et al. [30].

For all measurements, inter-assay and intra-assay coefficient of variation (CV) was less than 8% and 5%, respectively. Intra-assay CV was 1.8–3.8% and inter-assay CV was 4.6–7.9% for FGF23; according to the manufacturer’s report, the limit of detection was 5 pg/mL, and the limit of quantification was 6.5 pg/ mL.

### Statistical analysis

The results are expressed as means ± SD. In healthy subjects, the median and range of intact FGF23 plasma concentration and serum phosphate concentration were also assessed. Comparison of the data between healthy infants, prepubertal, pubertal, and postpubertal subjects was assessed by ANOVA with Tukey–Kramer multiple comparisons test. Repeated data were compared with the non-parametric Wilcoxon test. Comparison of intact FGF23 plasma concentration and serum phosphate concentration between healthy subjects and patients with XLH according to chronological age and pubertal development was performed using the non-parametric Wilcoxon's (Mann–Whitney) rank sum test. In both healthy subjects and patients with XLH, simple correlation analyses were carried out between intact FGF23 plasma concentration, serum phosphate concentration, and chronological age. In healthy postpubertal females, simple correlation analysis between intact FGF23 plasma concentration and time after menarche was assessed. Moreover, in patients with XLH, simple correlation analyses were carried out among TmP/GFR values and serum concentration of ALP, PTH, or 1,25(OH)_2_D. All statistical analyses were carried out using the SPSS (Statistical Package of Social Sciences, Chicago, IL, USA) for Windows software program. A *P* value ≤ 0.05 was considered significant.

## Results

### Intact FGF23 plasma concentration and serum phosphate concentration in healthy subjects

Mean ± SD, median, and range of intact FGF23 and serum phosphate concentrations in the healthy subjects according to chronological age and pubertal development are summarized in Table 1.

**Table 1:** Intact FGF23 plasma concentration and serum phosphate concentration in healthy subjects

Biochemical parameter	Infants (*n* = 30)	Prepubertal (*n* = 147)	Pubertal (*n* = 59)	Postpubertal (*n* = 46)
Intact FGF23				
Mean ± SD, pg/mL	49.7 ± 20.4	34.9 ± 12.6	47.2 ± 15.4	39.4 ± 16.5
Median, pg/mL	45.1	32.6	45.6	38.1
Range, pg/mL	11.2–90.3	16.5–75.8	19.8–91.1	15.6–87.3
Serum phosphate				
Mean ± SD, mmol/L	1.87 ± 0.19	1.57 ± 0.20	1.41 ± 0.20	1.16 ± 0.16
Median, mmol/L	1.87	1.55	1.39	1.16
Range, mmol/L	1.32–2.19	1.26–2.13	0.97–1.77	0.87–1.52

**Fig. 1:**
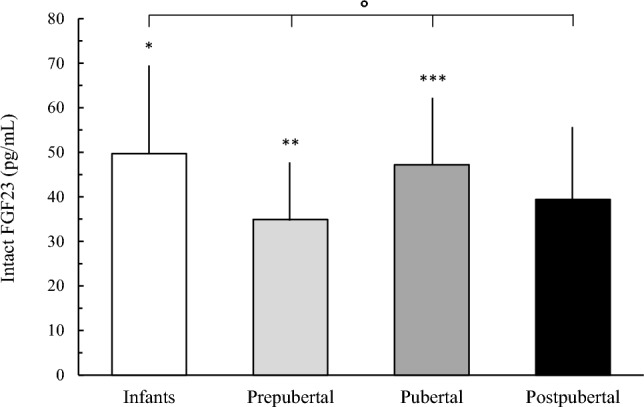
Intact FGF23 plasma concentration in infants, prepubertal, pubertal, and postpubertal healthy subjects. *P* < 0.0001 among the groups. *P < 0.001 vs prepubertal, *P* = NS vs pubertal, and *P* < 0.05 vs postpubertal. **P < 0.001 vs pubertal and *P* = NS vs postpubertal. ***P < 0.05 vs postpubertal

Intact FGF23 plasma concentration differed significantly (*P* < 0.0001) among the age groups; they were significantly higher in infants in comparison with prepubertal and postpubertal subjects but did not differ (*P* = NS) between infants and pubertal subjects. The pubertal subjects showed higher values (*P* < 0.05) compared with postpubertal subjects (Fig. 1). There was no difference in intact FGF23 plasma concentration between pubertal females who had not reached menarche yet (*n* = 9) and pubertal females who have reached menarche (*n* = 29) (38.0 ± 14.0 pg/mL and 48.4 ± 16.0 pg/mL, *P* = NS, respectively), whereas intact FGF23 plasma concentration was significantly higher in pubertal females who have had menarche than in postpubertal females (*n* = 22, 36.3 ± 11.4 pg/mL, *P* < 0.01).

**Fig. 2:**
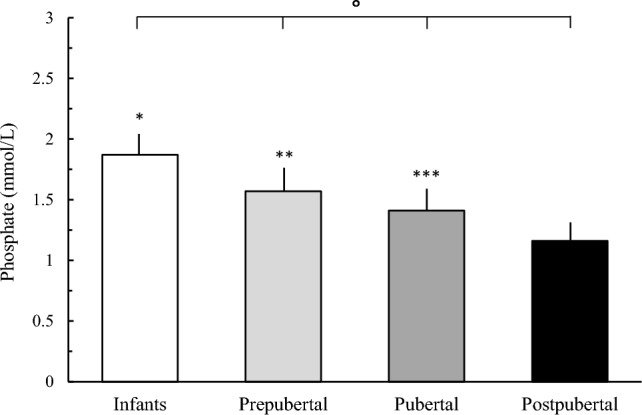
Serum phosphate concentration in infants, prepubertal, pubertal, and postpubertal healthy subjects. *P < 0.001 vs pubertal, pubertal, and postpubertal. **P < 0.001 vs pubertal and postpubertal. ***P < 0.001 vs postpubertal

Serum phosphate concentration progressively declined with age and was significantly (*P* < 0.0001) different among the groups. Serum phosphate concentration was significantly higher (*P* < 0.001) in infants in comparison with prepubertal, pubertal, and postpubertal subjects. Prepubertal subjects showed higher concentration (*P* < 0.001) compared with pubertal and postpubertal subjects, and pubertal subjects had higher concentration (*P* < 0.001) than postpubertal subjects (Fig. 2).

No difference (*P* = NS) between sexes was found in age and pubertal stage for both intact FGF23 plasma concentration and serum phosphate concentration (data not shown).

There was no correlation between intact FGF23 plasma concentration and serum phosphate concentration, and between intact FGF23 plasma concentration and chronological age or time after menarche in postpubertal females (data not shown). Serum phosphate concentration was inversely correlated with age (*r* = −0.72, *P* < 0.0001).

### Clinical and biochemical data in patients with XLH

**Fig. 3:**
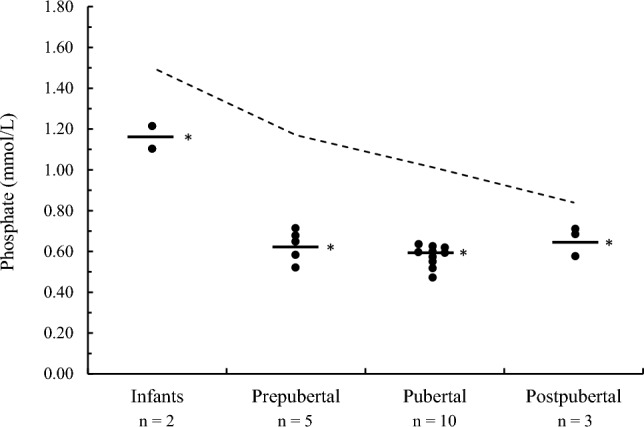
Mean and individual values of serum phosphate concentration in patients with XLH according to chronological age and pubertal development compared with the lower limit of normal (−2 SD, broken line) assessed in the healthy subjects of the present study. *P < 0.0001 between patients and healthy subjects

Clinical and biochemical data in patients with XLH at entry into the study, and *PHEX* gene mutation, are summarized in Table 2. All patients showed hypophosphatemia, reduced TmP/GFR, and increased ALP concentration. Mean serum phosphate concentration was significantly lower (*P* < 0.0001) in patients with XLH in comparison with healthy subjects according to chronological age and pubertal development: infants 1.16 ± 0.05 mmol/L, *n* = 2; prepubertal 0.62 ± 0.10 mmol/L, *n* = 5; pubertal 0.58 ± 0.05 mmol/L, *n* = 10; postpubertal 0.63 ± 0.06 mmol/L, *n* = 3; all the patients showed reduced serum phosphate concentration compared with the lower limit of healthy subjects (Fig. 3).

**Table 2:** Clinical and biochemical data in patients with XLH at entry into the study, and *PHEX* gene mutation

Case	Sex	Age at diagnosis, (years)	Age at entry (years)	Length/height^b^ (*Z*-score)	Serum P, (mmol/L)	TmP/GFR (mg/dL)	Serum ALP (IU/L)	Serum PTH, (pg/mL)	Serum 1,25(OH)_2_D, (pg/mL)	*PHEX *gene mutation
1	M	0.08	0.08^a^	−1.7	1.21	3.7	586	47.0	23.2	Fc.1255G > T
2	M	0.1	0.2	−2.0	1.10	3.2	724	31.4	12.2	c.254G > T
3	M	0.1	12.0	−2.3	0.58	1.3	550	33.6	24.1	Deletion ex12
4	M	0.1	14.1	−2.2	0.52	1.7	589	51.5	32.2	c.1645C > T
5	M	0.3	4.6	−2.6	0.68	1.8	554	39.5	34.3	c.1529G > C
6	F	1.0	17.5	−1.6	0.67	1.6	199	69.2	26.0	Fc.1255G > T
7	F	1.1	8.3	−3.4	0.48	1.4	463	26.9	38.7	c.1643T > C
8	F	1.4	17.8	−2.2	0.68	2.0	221	46.8	24.7	c.1616_1617delCC
9	F	1.7	9.8	−2.1	0.47	1.8	578	40.6	19.8	c.1092C > A
10	F	1.8	11.0	−1.8	0.62	1.9	547	54.2	02.2	c.2239C > T
11	F	2.1	2.1	−2.1	0.73	1.6	649	44.3	25.5	c.1586 + 1G > A
12	F	2.4	2.8	−2.5	0.53	1.2	908	38.1	23.0	deletion ex15
13	F	2.4	8.2	−1.1	0.69	1.9	401	49.7	26.7	c.1079 + 5G > A
14	F	2.5	9.9	−3.2	0.63	1.2	531	48.4	36.2	c.2053_2054insCTTCT
15	M	3.5	13.3	−3.4	0.52	1.8	604	38.6	21.7	c.1041_1042insT
16	M	3.6	15.1	−1.2	0.65	1.9	450	37.5	27.5	c.2239C > T
17	M	4.4	16.3	−2.6	0.55	1.4	396	43.6	14.7	c.118 + 1G > A
18	M	5.5	9.3	−2.3	0.59	1.4	477	55.7	33.5	c.1061C > T
19	F^c^	5.9	15.7	−3.0	0.57	1.5	465	52.8	29.4	Deletion ex4-22
20	F^c^	5.9	15.7	−3.1	0.61	1.7	494	58.9	36.0	Deletion ex4-22
Mean ± SD		2.3 ± 1.9	10.2 ± 5.6	−2.3 ± 0.7	0.65 ± 0.18	1.8 ± 0.6	519.3 ± 152.8	43.6 ± 9.6	25.9 ± 7.0	
Normal values					d	e	f	8–40	25–110	

Familial form: case 1–7, 9, 10, 13, 16, and 18; sporadic form: case 8, 11, 12, 14, 15, 17, 19, and 20

^a^Untreated at entry

^b^Length in patients 1–10; height in patients 11–20

^c^Homozygous twins

^d^Present study: see Table 1

^e^ < 2 years 5.1 ± 0.5 mg/dL; 2–12 years 4.6 ± 0.6 mg/dL; 12–16 years 4.1 ± 0.6 mg/dL [29]

^f^Upper limit of normal: infants, 450 IU/L (case 1 and 2); prepubertal, 300 IU/L (case 5, 7, 12, and 13); pubertal, 390 IU/L (3, 4, 9, 10, 14–16, 18–20); postpubertal, 180 IU/L (cases 6, 8, and 17)

Serum PTH concentration was normal (≤ 40 pg/mL; 30.2 ± 11.7 pg/mL) in 7 patients (35%) and slightly increased (> 40 pg/mL; 49.5 ± 7.6 pg/mL) in 13 patients (65%). There was no difference (*P* = NS) in intact FGF23 plasma concentration between patients with normal serum PTH concentration and those with increased serum PTH concentration (145.6 ± 88.2 pg/mL and 136.2 ± 125.0 pg/mL, respectively). Serum 1,25(OH)_2_D concentration was reduced (< 25 pg/mL; 20.4 ± 4.3 pg/mL) in 8 patients (40%) and in the normal range (≥ 25 pg/mL; 30.8 ± 4.5 pg/mL) in 12 patients (60%). Intact FGF23 plasma concentration was significantly higher (*P* < 0.01) in patients with reduced serum 1,25(OH)_2_D concentration than in patients with serum 1,25(OH)_2_D in the normal range (219.0 ± 142.1 pg/mL and 86.5 ± 31.6 pg/mL, respectively).

**Fig. 4:**
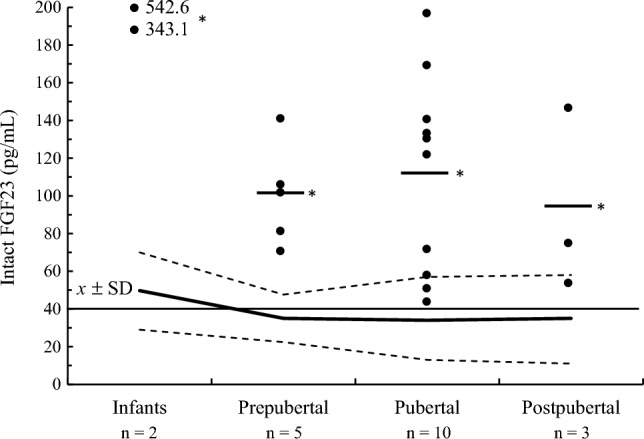
Mean and individual values of intact FGF23 plasma concentration in patients with XLH according to chronological age and pubertal development compared with data obtained in healthy subjects of the present study expressed as mean ± SD (bold and broken lines, respectively). Continuous thin line indicates intact FGF23 concentration of 40 pg/mL. *P < 0.0001 between patients and healthy subjects

Mean intact FGF23 plasma concentration was significantly (*P* < 0.0001) higher in infant, prepubertal, pubertal, and postpubertal patients with XLH in comparison with healthy subjects according to chronological age and pubertal development (Fig. 4). Infant patients with XLH had the highest intact FGF23 plasma concentration of the enrolled groups of patients. All but three patients with XLH had a value of intact FGF23 above the upper limit of healthy subjects; in all patients, intact FGF23 plasma concentration was above the value of 40 pg/mL (Fig. 4).

Intact FGF23 plasma concentration was inversely correlated with serum phosphate concentration, TmP/GFR values, and serum 1,25(OH)_2_D concentration; serum phosphate concentration and TmP/GFR values were positively correlated; there was no correlation between serum phosphate concentration and serum 1,25(OH)_2_D concentration (Table 3). There was no correlation between intact FGF23 plasma concentration and chronological age, serum PTH or ALP concentrations (data not shown), and between serum 1,25(OH)_2_D concentration and TmP/GFR values (data not shown).

**Table 3:** Correlation coefficients among intact FGF23 plasma concentration, serum phosphate concentration, serum 1,25(OH)_2_D concentration, and TmP/GFR values in patients with XLH

Biochemical parameters	Plasma intact FGF23		Serum phosphate		Serum 1,25(OH)_2_D		TmP/GFR	
	*r*	*P*	*r*	*P*	*r*	*P*	*r*	*P*
Intact FGF23	–		−0.65	< 0.01	−0.51	< 0.05	−0.83	< 0.0001
Serum phosphate	–		–		0.14	NS	0.66	< 0.01

## Discussion

In healthy subjects, intact FGF23 plasma concentration differed according to chronological age and pubertal development. Age of menarche and time after menarche did not affect intact FGF23 plasma concentration. In patients with XLH, del Pino et al. [31] showed that the onset of puberty and the age of menarche were no different from the general population, indicating that they were not confounding factors for the assessment of intact FGF23 plasma concentration.

Highest FGF23 concentration occurred during infancy and puberty when growth velocity is maximal [24]. A similar pattern was found by Fischer et al. [17] by measuring the C-terminal fragment of FGF23 molecule but not in other studies assessing intact or C-terminal FGF23 molecule [18, 20]. In accordance with some studies [18–20], but not with others [17], we did not find a correlation of FGF23 concentration with age. In our cohort of healthy subjects, there was no difference of intact FGF23 plasma concentration with regard to sex, as found by Stanczyk et al. [20]; whereas, the concentration of C-terminal FGF23 was higher in girls than in boys in the study by Gkentzi et al. [18].

In healthy subjects, serum phosphate concentration progressively declined from infancy to postpuberty confirming well-known data [32–34]; they were not correlated with intact FGF23 plasma concentration, as found by Mitchell et al. in girls [19]. Conversely, a positive correlation between serum phosphate concentration and C-terminal or intact FGF23 concentration was shown in some studies [17, 18]. Likely, the total number and range of age, pubertal development, and the assay used to assess FGF23 concentration were the main determinants of the contrasting data reported in various studies.

Physiological changes of intact FGF23 and phosphate concentrations in infants, children, and adolescents should be taken into account for a correct interpretation in patients with hypophosphatemic disorders. During infancy, both healthy subjects and patients with XLH had higher intact FGF23 plasma concentration compared with children and adolescents. However, the patients with XLH showed higher mean intact FGF23 plasma concentration in comparison with healthy subjects at any age.

The majority of patients with XLH had increased intact FGF23 plasma concentration and only three patients showed a value in the upper limit of normal. Nevertheless, intact FGF23 plasma concentration was above 40 pg/mL in all the patients with XLH we examined. Endo et al. [35, 36] reported that serum phosphate concentration less than 1.40 mmol/L for infants and less than 1.09 mmol/L for children and a value of intact FGF23 over 30 pg/mL, assessed by ELISA assay (Kainos, Japan), could be a main criteria for the biochemical diagnosis of XLH. In our cohort of patients with XLH mean serum phosphate concentration was lower (infants 1.16 mmol/L; prepubertal 0.62 mmol/L) compared with that reported by Endo et al. [35] and the cutoff of intact FGF23 was above 40 pg/mL. This different cutoff may be due to some differences in assay calibration or sample collection for assessing FGF23 concentration being plasma EDTA and serum for Liaison and Kainos assay, respectively. Shimizu et al. [37] proposed the extrapolated cutoff of 25 pg/mL based on the coefficients of variation to discriminate between FGF23-related and FGF23 not-related hypophosphatemic disorders. These data suggest that the values of circulating intact FGF23 are method dependent so that appropriate reference values should be used for the diagnosis in patients with hypophosphatemic disorders.

The lack of correlation between the intact FGF23 and phosphate concentration we found in healthy subjects suggested that intact FGF23 concentration is not regulated by circulating phosphate. Conversely, intact FGF23 was inversely correlated with phosphate concentration and TmP/GFR values in patients with XLH, supporting a main role of FGF23 excess in impairing the regulation of phosphate metabolism.

In patients with XLH, FGF23 excess was associated with low or inappropriately normal serum 1,25(OH)_2_D concentration for the degree of hypophosphatemia [7, 13]. Experimental studies in mice showed that the administration of 1,25(OH)_2_D increased FGF23 concentration, while the disruption of 1,25(OH)_2_D pathways reduced circulating FGF23, suggesting that FGF23 acts as a vitamin D counterregulatory hormone [38, 39]. On the other hand, it has been shown that conventional treatment in patients with XLH further increased FGF23 concentration, which supports the possibility that this may limit the effectiveness of conventional therapy [22, 40]. Intact FGF23 plasma concentration was inversely correlated with serum 1,25(OH)_2_D concentration in the cohort of patients with XLH we examined, and the patients with reduced serum 1,25(OH)_2_D concentration had higher intact FGF23 plasma concentration than the patients in whom serum 1,25(OH)_2_D concentration was in the normal range. This may support the evidence that 1,25(OH)_2_D deficiency is the result of impaired synthesis/catabolism induced by FGF23 excess. According to this hypothesis serum 1,25(OH)_2_D concentration increased significantly after the inhibition of FGF23 in patients with XLH receiving burosumab treatment, which probably reflected the enhanced conversion of 25-hydroxyvitamin D to 1,25(OH)_2_D [41–43].

Some data showed that FGF23 inhibits PTH synthesis and secretion directly through the MAPK pathway [44, 45]. Moreover, FGF23 may regulate PTH concentration through the FGF receptor but only in normocalcemia [46]. Patients with XLH usually have normal or slightly increased serum PTH concentration [7, 12, 13], as found in our patients. Carpenter et al. [40] showed that intact FGF23 and mid-molecule PTH concentration were positively correlated in patients with XLH during conventional treatment, suggesting that the propensity to develop secondary and even tertiary hyperparathyroidism may be due to a resistance to FGF23 at the level of the parathyroid cell, limiting FGF23’s ability to inhibit PTH secretion. We did not find a correlation between intact FGF23 and intact PTH concentrations in our patients, and intact FGF23 plasma concentration did not differ between the patients with normal or increased serum PTH concentration. Indeed, the assays measuring intact PTH molecule have increased sensitivity, enhanced precision, and improved specificity in comparison with PTH assays measuring the biologically inactive mid-molecule and carboxyl-terminal fragments of the hormone [47]. Moreover, secondary or tertiary hyperparathyroidism may be related to the duration of the conventional treatment and to oral inorganic phosphate salts supplement [12, 13, 48]. Therefore, conventional treatment could be a main determinant in affecting the correlation between circulating PTH and FGF23.

A limiting factor of the present study could be the small number of the healthy subjects examined in each considered category and of patients with XLH. However, the results were quite consistent and were obtained after an accurate selection of healthy subjects.

In conclusion, our study showed that chronological age and pubertal development, but not sex and age or time after menarche, were main determinants of intact FGF23 plasma concentration in healthy subjects. Infancy and puberty were characterized by the highest intact FGF23 plasma concentration. Serum phosphate concentration declined with age and was different between the pediatric ages. Physiological changes in serum phosphate and intact FGF23 plasma concentrations should be taken into account in the clinical setting. Patients with XLH had higher intact FGF23 plasma concentration than healthy subjects according to chronological age and pubertal development. Infant patients showed the highest circulating intact FGF23 plasma concentration. In addition to clinical signs, hypophosphatemia associated with intact FGF23 plasma concentration greater than 40 pg/mL, assessed by the Liaison® XL assay, may be a crucial marker for the early diagnosis of XLH in pediatric patients.

## Data Availability

Data are available on request, only if appropriate and with the permission of the subject.
